# Virtual and Reality: A Neurophysiological Pilot Study of the Sarcophagus of the Spouses

**DOI:** 10.3390/brainsci13040635

**Published:** 2023-04-07

**Authors:** Andrea Giorgi, Stefano Menicocci, Maurizio Forte, Vincenza Ferrara, Marco Mingione, Pierfrancesco Alaimo Di Loro, Bianca Maria Serena Inguscio, Silvia Ferrara, Fabio Babiloni, Alessia Vozzi, Vincenzo Ronca, Giulia Cartocci

**Affiliations:** 1 Unit of Histology and Medical Embryology, SAIMLAL Department, Sapienza University of Rome, 00185 Rome, Italy; andrea.giorgi@uniroma1.it (A.G.); alessia.vozzi@uniroma1.it (A.V.); 2 BrainSigns Ltd., 00185 Rome, Italy; stefano.menicocci@brainsigns.com (S.M.); biancams.inguscio@uniroma1.it (B.M.S.I.); silvia.ferrara@brainsigns.com (S.F.); fabio.babiloni@uniroma1.it (F.B.); vincenzo.ronca@uniroma1.it (V.R.); 3 Department of Molecular Medicine, Sapienza University of Rome, 00161 Rome, Italy; 4 Department of Classical Studies, Duke University, Durham, NC 27708, USA; maurizio.forte@duke.edu; 5 Art and Medical Humanities Lab, Sapienza University of Rome, 00185 Rome, Italy; vincenza.ferrara@uniroma1.it; 6 Department of Political Sciences, Roma Tre University, 00145 Rome, Italy; marco.mingione@uniroma3.it; 7 Department of Law, Economics, Politics and Modern Languages, Libera Università Maria SS. Assunta (LUMSA), 00192 Rome, Italy; p.alaimodiloro@lumsa.it; 8 Department of Human Neuroscience, Sapienza University of Rome, 00185 Rome, Italy; 9 Department of Computer Science, Hangzhou Dianzi University, Hangzhou 310018, China; 10 Department of Computer, Control and Management Engineering “Antonio Ruberti”, Sapienza University of Rome, 00185 Rome, Italy

**Keywords:** neuroaesthetics, frontal alpha asymmetry, frontal theta, emotion, virtual reality, archaeology

## Abstract

Art experience is not solely the observation of artistic objects, but great relevance is also placed on the environment in which the art experience takes place, often in museums and galleries. Interestingly, in the last few years, the introduction of some forms of virtual reality (VR) in museum contexts has been increasing. This has solicited enormous research interest in investigating any eventual differences between looking at the same artifact either in a real context (e.g. a museum) and in VR. To address such a target, a neuroaesthetic study was performed in which electroencephalography (EEG) and autonomic signals (heart rate and skin conductance) were recorded during the observation of the Etruscan artifact “Sarcophagus of the Spouses”, both in the museum and in a VR reproduction. Results from EEG analysis showed a higher level of the Workload Index during observation in the museum compared to VR (*p* = 0.04), while the Approach–Withdrawal Index highlighted increased levels during the observation in VR compared to the observation in the museum (*p* = 0.03). Concerning autonomic indices, the museum elicited a higher Emotional Index response than the VR (*p* = 0.03). Overall, preliminary results suggest a higher engagement potential of the museum compared to VR, although VR could also favour higher embodiment than the museum.

## 1. Introduction

“Art experience means the rich experience of artistic objects that are mostly embedded in situational, social, and cultural contexts: for instance when encountering art in art galleries or museums” [1]. Such a statement stresses the relevance of the environment in which the art experience takes place, mentioning “traditional” contexts like galleries and museums. However, this notion needs to take into account the widening development of the introduction of virtual reality (VR) in museum contexts, which often regard such technology as a novel way for improving the appeal and enjoyment of the fruition of their collections [2], ultimately attracting more visitors [3]. It has been reported that in Europe, about 35% of museums have already introduced some form of 3D presentation of objects [4]. The same authors also reported that relative to archaeological contexts, the opinion of museum professionals and other key experts highlighted great enthusiasm for 3D and awareness of its potential, with around 65% of archaeological museums affirming that 3D had an “important” or “very important” role to play in presenting archaeology to the public and in the study of material culture [4]. Such interest even led to the definition of best practices for the construction of virtual museums [5] and to the development of dedicated systems, enabling the curators to set up virtual museum exhibitions [6]. Of course, such innovations would strongly contribute to the dissemination of artistic knowledge and enjoyment and to the cultural heritage theme, but the spontaneous questions rising from this scenario are: could a VR experience replace a real experience of an artifact? What are we gaining and what are we loosing? Is VR a hyperinformative space?

In order to respond to such questions, help comes from a widening novel research area, combining the notions of art experience with neuroscientific methods: neuroaesthetics. It was founded a few decades ago by Semir Zeki [7,8] and investigates the neurobiological correlations of the aesthetic experience [9,10]. Neuroaesthetics has already helped in digging into controversial and complex themes linked to Art fruition, as in the case of the use of artificial intelligence in art. Indeed, it has allowed the disentanglement of declarative prejudice from physiological reactions to items perceived as different from the traditional form, for abstract paintings [11] and also in investigating cultural influences on such judgements [12].

There are few previous studies concerning the comparison between the art experience in VR and real environments and they do not include all cerebral, declarative and autonomic responses. One of those studies, aimed at investigating the elicitation of the same emotional response based on both electroencephalographic (EEG) and electrocardiographic biosignals recorded during the free exploration of an art museum and its 3D immersive reconstruction, found 95.27% accuracy for the real vs. virtual classifier using only EEG mean phase coherency features [13]. Furthermore, it was evidenced self-reported psychological arousal for both real and VR museums, but only in the real one were reported differences in terms of cardiovascular responses, suggesting that the VR reconstruction of a real environment might be self-reported as psychologically arousing but might not necessarily evoke the same cardiovascular changes as a real arousing elicitation [14].

As already mentioned, the introduction of VR in archaeology may be of particular importance for the preservation of ancient artifacts. This is the reason for the choice of employing as a stimulus the famous Etruscan terracotta funerary artifact at Villa Giulia National Etruscan Museum in Rome: “The Sarcophagus of the Spouses”. This artifact dates to the period between 530 and 520 BC and reproduces a banquet with a couple in a half-reclined position.

The objective of comparative research between a virtual and an actual experience of the same object is to examine the neuroaesthetic experience and visual impact among various types of users/visitors. In the VR experience of the Sarcophagus of the Spouses, the experiment centered on comparing actual and empirical observations and determining the role of VR in the art experience process. Furthermore, where real visits are feasible, a digital model would be valuable for sufficiently informing the observer prior to a museum visit or, alternatively, for creating a post-empirical experience. The sustained activation of the brain representation of motor information in the absence of movement constitutes the experiential foundation of what we see or imagine perceiving. This enables immediate perception of the relational nature connecting space, things and other people’s activities to our bodies. Thus, the analysis of archeological records, particularly when acquired in 3D and enabling a true immersive interaction, naturally lends itself to a ‘performative’ study of the acquired evidence, enabled by the possibility of empirically documenting the relationship between a given object, be it a wall or a depiction or a room, and the body’s activity, practices and habits it evokes by means of the embodied simulation triggered by its visualization or haptic exploration. The comparison between the responses evoked by physical environments and their virtual simulations has been studied to some degree through the assessment of psychological responses [15], cognitive performance [16] and—to a much lesser extent—physiological and behavioural responses [13,17,18]. Given the well-known poor sensitivity of declarative evaluations or self-assessments [19,20], it appears extremely relevant to investigate the neurophysiological underpinnings of people’s reactions to an art experience in both VR and a real context along with the implicit demand posed by the rapid development of our societies and for the concern about the modality of younger people learning ancient culture and art, finally supporting the design of museum environments and inclusivity.

Aim of the present pilot study, which is part of the international project NeuroARTifact (https://neuroartifact.org, accessed on 28 March 2023), was to investigate the declarative and biosignal-based reactions to the observation of the same artifact in both VR and a real museum environment. As a guide for the investigation, the following research questions were asked:

RQ1: What does the vision of the real Sarcophagus of the Spouses in the museum arouse compared to its reproduction in VR?

RQ2: How does the art experience differ over time in the museum or laboratory conditions?

RQ3: Is there a relationship between unconscious and self-reported perception?

## 2. Materials and Methods

### 2.1. Participants

A total of 50 participants were initially enrolled in the study, but due to several drop-outs, only 26 participants (16 females, 10 males; mean age = 28.80, SD = 11.08) took part in both sessions (museum and laboratory) and therefore were actually included in the pilot study. All participants were students or researchers recruited as volunteers from the University of Rome “La Sapienza”. Inclusion criteria were the absence of expertise in archaeology or VR technologies, absence of major neurological and psychiatric pathologies, normal or corrected vision and age between 18 and 45 years old. The order of the experimental observation (museum or laboratory) was randomly assigned to participants, resulting in 18 participants (10 females, 8 males; mean age = 30.76, SD = 12.78) first exposed to the artwork in a real context (museum) and then in a virtual context (laboratory) and 8 participants (6 females, 2 males; mean age = 25.14, SD = 5.80) first exposed to the artwork in a virtual context (laboratory) and then in a real context (museum).

Informed consent was obtained from all participants prior to the start of the experimental sessions. All participants were informed that they would have to conduct two separate sessions: one at the Art and Medical Humanities Lab at the Faculty of Pharmacy and Medicine, University of Rome “La Sapienza”, and the other at the National Etruscan Museum of Villa Giulia. Recordings were made on two separate days. Participants were not informed which artwork they were going to see nor that they would see the same artwork in two different contexts. We asked participants not to tell others what they saw until after they completed all stages of the research. Participants did not report any motion sickness and, in general, no kind of discomfort related to the VR condition.

### 2.2. Materials

The stimulus used for the research was the Sarcophagus of the Spouses preserved at the ETRU National Etruscan Museum of Villa Giulia and Villa Poniatowski in Rome. We created a virtual simulation of the museum experience where participants could observe the octagonal room in which the sarcophagus is placed. Each participant was free to visually explore the virtual room as well as the real room. We used a 3D model reproduction of the sarcophagus made by the Visual Computing Lab ISTI-CNR (http://vcg.isti.cnr.it/sposi/, accessed on 28 March 2023), which scanned the real sarcophagus to create a highly realistic model. The museum room was replicated through Blender 3D, a 3D polygonal modelling software. Both the model and the room were assembled and rendered for virtual reality by SightLab_VR software (owned by WorldViz). Simulations in VR employed an HTC Vive Pro Eye headset.

### 2.3. Locations

The research was performed in two separate locations. The involved participants observed both the work in vivo and the sarcophagus in VR. Recordings of reactions to real artwork were performed at the Etruscan Museum of Villa Giulia. The recordings of reactions to the virtual artwork were performed at the Art and Medical Humanities Lab at the Faculty of Pharmacy and Medicine, University of Rome “La Sapienza”.

### 2.4. Protocol

In order to standardize the protocol and the stimulus presentation, participants were instructed to visually explore the environment (both in the laboratory and museum). They were not allowed to walk or to touch the exhibit and they had to limit communication with the researchers as much as possible.

Upon arrival, participants were asked to carefully read and sign the informed consent module (complies with General Data Protection Regulations—GDPR—European Union—2016/679). If participants agreed to participate, the collection of data started. First, participants filled out a brief questionnaire to collect information about their gender, age and course of studies. After these preliminary activities, they were equipped with the EEG and EDA/HR recording devices. Before proceeding to the experimental task, collection of baseline neurophysiological signals was performed. Participants were asked to close their eyes for a minute in order to collect cortical signals and to compute the Individual Alpha Frequency (IAF [21]). After this, they were asked to look at a plain wall for a minute in order to record their resting state activity, to be used as a reference condition in the analysis. In the laboratory, they were presented with a virtual environment identical to the real one used for the resting state recording in the museum. After this task, participants were placed in front of the sarcophagus with closed eyes; they were then instructed to open their eyes and the biosignal recording started. The observation task lasted one minute, and it was always performed from the same observation point both in virtual and real environments.

**Figure 1:**
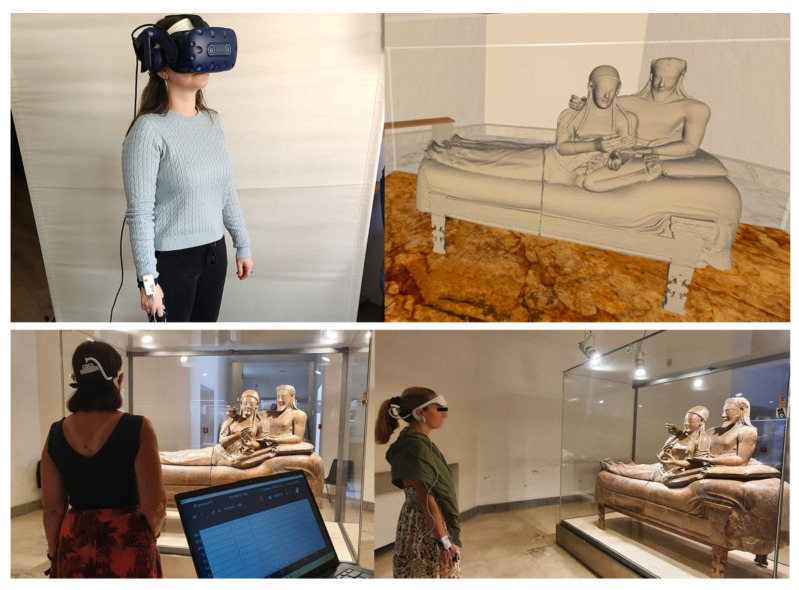
(top—left) example of a participant in the laboratory wearing the HTC Vive Pro Eye headset, EEG Revive headset, Shimmer on the right wrist and sensors for autonomic biosignal recordings on fingers; (bottom) example of two participants undergoing the experimental session in the museum, wearing Revive headset, Shimmer on the right wrist and sensors for autonomic biosignal recordings on fingers; (bottom—left) the recording laptop connected to Revive and Shimmer instruments, showing the biosignal traces (permission to show the Sarcophagus of the Spouses courtesy of the ETRU National Etruscan Museum of Villa Giulia in Rome).

The order of stimulus presentation (real/virtual) was pseudorandomized among participants. Half of the sample observed the artwork in the museum first while half observed the artwork in VR first (Figure 1).

### 2.5. Statistical Analysis

In order to measure and compare the implicit perception of the two conditions (Location factor, two levels: Museum and Laboratory), neurophysiological signals were used to compute three different indicators of the participants’ mental state: the Approach–Withdrawal Index [11], Workload Index [22,23] and Emotional State Index [24,25]. At the same time, both after the observation of the sarcophagus and its virtual reproduction, participants were asked to rate their explicit impressions about valence, arousal, familiarity, liking and beauty. Explicit impressions were collected at the end of the observation; therefore, only one point in time was detected. On the contrary, implicit perception recording, i.e., mental state assessment, was performed continuously during the observation, and for this reason, we performed the analysis on the entire recording (one minute long) and on both the initial part (first 10 s) and final part (last 10 s) of the observation (Time factor, levels: “All”; or alternatively “Beginning” and “End”). 

When comparing two conditions, the Shapiro-Wilk test of normality was conducted on the data distribution prior to doing a two-tailed paired Student’s t-test (in the case that normality was confirmed) or a Wilcoxon test (in the case that normality was rejected). A repeated-measures ANOVA, followed by a related Duncan’s post hoc test or a Friedmann nonparametric test, was conducted when comparing multiple conditions.

### 2.6. Biosignal Recording and Processing

#### 2.6.1. EEG Recording and Analysis for Workload and Approach–Withdrawal Assessment

Electroencephalography (EEG) monitoring was performed using the Revive device (https://brainsigns.com/it/component/k2/mostra-adi-design-index-2021-esposizione-headset-revive; https://www.adi-design.org/2021_l00859, both websites accessed on 28 March 2023), designed and produced by BrainSigns s.r.l. (Italy, Rome), a spin-off of the University of Rome “La Sapienza”, in collaboration BrainProducts GmbH (Gilching, Germany). The device is easy to fit and comfortable. It features 8 frontal electrodes (AFz, AF3, AF4, Fz, F3, F4, F5, F6) placed according to the 10–10 International System and a reference and ground electrode placed each on one of the mastoids.

The device has been validated and is capable of recording the EEG signal extremely accurately [26]. The sampling frequency was 250 (Hz). A 50 Hz notch filter was applied for removing main line power interference. The EEG recordings were also band-pass filtered [low-pass filter cut-off frequency: 40 (Hz), high-pass filter cut-off frequency: 2 (Hz)] and then the Independent Component Analysis (ICA) was used to remove eyeblinks and muscular artefacts. For other sources of artefacts, specific algorithms of the EEGLAB toolbox [27] were applied. Specifically, the ICA-processed signal was then separated into 1 s long epochs and three criteria were applied in order to automatically recognize artefactual data. Firstly, EEG epochs with a signal amplitude exceeding ±80 mV (Threshold criterion) were marked as ‘‘artefacts’’. Then, each EEG epoch was interpolated in order to check the slope of the trend within the considered epoch (Trend estimation). If the slope was higher than 20 mV/s, the considered epoch was marked as an artefact. Finally, the signal sample-to-sample difference (sample-to-sample criterion) was analysed. If such a difference, in terms of absolute amplitude, was higher than 25 mV, i.e., an abrupt variation (not physiological) happened, the EEG epoch was marked as an artefact. In the end, the EEG epochs marked as artefacts were removed from the EEG dataset with the aim of having a clean EEG signal to perform the analyses.

From the artefact-free EEG, the Global Field Power (GFP) was calculated for the EEG frequency bands of interest for the mental state of interest: Alpha and Theta. The GFP was chosen as the parameter of interest describing brain EEG activity since it has the advantage of representing, in the time domain, the degree of synchronization or a specific cortical region of interest in a specific frequency band [28,29,30]. The Alpha band was defined according to the Individual Alpha Frequency (IAF) value [21] computed for each participant. Since the Alpha peak is mainly prominent during rest conditions, the subjects were asked to keep their eyes open for a minute before starting the experiment. Such a condition was then used to estimate the IAF value specifically for each participant. Consequently, an EEG ‘‘strict’’ Alpha band was defined as Alpha = (IAF − 1):(IAF + 1) Hz. This definition of the Alpha band is more restrictive (thus, ‘‘strict’’) compared to the vast majority of Alpha band definitions that can be found in the scientific literature, which is (IAF-2):(IAF + 2) Hz. This approach was selected according to Klimesch [31], who demonstrated that a tighter band around the IAF can be considered as Alpha to avoid the impact from closer EEG frequency band (Theta and Beta) variations on the observed phenomena in the Alpha band. The GFP was calculated over all EEG frontal channels for each epoch using a Hanning window of the same length as the considered epoch (1 s length, which means 1 Hz of frequency resolution).

The EEG Workload Index represents the mental resources allocated to perform a task or process a stimulus. Several studies have reported increased Theta activity in frontal regions with increased workload demanded by a task [22,23,32,33]. This augmented Theta activity reflects increased activation of the prefrontal cortex area, which is involved in this higher-level cognitive phenomena. In this view, to measured the Workload level during the observation of the sarcophagus and its VR reproduction, the Theta band value across every frontal electrode (AFz, AF3, AF4, Fz, F3, F4, F5 and F6) was averaged and used as a direct measure of workload, according to Formula (1):

(1) $$\frac{\sum_{i=1}^{n}Frontal\;Theta}{n}$$

where *n* is the number of frontal electrodes. The index was normalized by using the median and median absolute deviation of the baseline.

The index is based on the asymmetric response of the brain in the processing of pleasant and unpleasant stimuli. From the literature, different activation of the frontal region has been reported with respect to the motivation to withdraw from a stimulus, where an asymmetrical distribution of the Alpha band represents a tendency to reject and avoid the stimulus [29,32,34,35].

The Approach–Withdrawal Index was normalized by using the median and median absolute deviation of the baseline. It was then computed following Formula (2):

(2) $$\frac{\sum_{i=1}^{n}Frontal\;Alpha\;dx}{n}-\frac{\sum_{i=1}^{m}Frontal\;Alpha\;sx}{m}$$

where *n* is the number of frontal right electrodes and *m* is the number of frontal left electrodes.

The Alpha band on the right and left frontal region was calculated by averaging the value of, respectively, right (AF4, AF6 and F4) and left electrodes (AF3, AF5 and F3). Following this calculation, a higher value of the index represents a higher motivation to withdraw from a stimulus.

#### 2.6.2. Electrodermal Activity (EDA) and Heart Rate (HR) Recording and Analysis for Emotional State Assessment

The EDA was recorded using the Shimmer3+ GSR unit (Shimmer Sensing, Dublin, Ireland) with a sampling frequency of 64 Hz. Shimmer sensors were placed on the second and third fingers of the participant’s non-dominant hand. 

The EDA was firstly low-pass-filtered with a cut-off frequency of 1 Hz and then processed by using the Ledalab Suite [36], a specific open-source toolbox implemented within the MATLAB (MathWorks, Natik, Massachussets) environment for EDA processing (details in Table 1). A continuous decomposition analysis [37] was applied in order to estimate the tonic (Skin Conductance Level—SCL) and phasic (Skin Conductance Reaction—SCR) components [38]. The SCL is the slow-changing component of the EDA signal, mostly related to the global arousal of the participant. In contrast, the SCR is the fast-changing component of the EDA signal, usually related to single-stimuli reactions. The EDA components, as well as the other neurophysiological parameters, were estimated using both a 60 s time resolution and averaging within each experimental condition.

The Shimmer3+ device was also used to collect a photoplethysmographic signal (PPG) to derive the HR measure. The PPG signal was filtered using a 5th-order Butterworth band-pass filter (1–1 Hz and 1–4 Hz, respectively) in order to reject the continuous component and high-frequency interference, such as that related to the main power source (details in Table 1). Another purpose of this filtering was to emphasize the QRS complex of the ECG signal [39]. The following step consisted of computing the ECG (PPG) signal to the power of 3 to emphasize heartbeat peaks, as they generally have the highest amplitude and at the same time reducing spurious artefact peaks. Finally, the distance between consecutive peaks (i.e., each R peak corresponds to a heartbeat) was measured to estimate HR values every 60 s. The Pan–Tompkins algorithm [40] was used for HR estimation. A combination of HR and SCL measurements was adopted in order to estimate emotional state [41,42]. In this regard, an *Emotional Index* (EI) was defined as (3):

(3) $$Emotional\;Index=\left|SCL\right|*HR$$

where *SCL* and *HR* were normalized by subtracting the corresponding baseline and dividing by the corresponding standard deviation. The Emotional Index is presented as a fusion of *HR* and *SCL* measurements, respectively reflecting valence and arousal associated with the processing of a stimulus or the performance of a task [42]. We refer to the effects plane [41,43], where the *HR* (horizontal axis) and the GSR (vertical axis) serve as the coordinates for a point in this space. Previous research has shown that these two autonomic indicators connect with valence and arousal, respectively [24,44]. According to the EI interpretation, the higher the value, the more emotional involvement the subject has, and vice versa. The EI Index was normalized by using the median and median absolute deviation of the baseline. The combination of these two parameters was adopted because the sensitivity of this emotional index has already been described in previous works [42]. 

### 2.7. Behavioural Data

After the observation of the sarcophagus and its virtual reproduction, participants were asked to rate, on a scale from 1 to 7, their explicit impressions about valence, arousal, familiarity, liking and beauty. Explicit impressions were collected at the end of the observation; therefore, just one point in time was detected.

## 3. Results

### 3.1. EEG-Based Indices

By means of the neurophysiological assessment, we aimed to answer RQ1 and RQ2. We were therefore interested in highlighting eventual differences in the mental state induced by the two different types of observations and investigating the temporal dynamics of the perception, i.e., if there is a difference in perception, is it already visible in the very first seconds? On the contrary, do these differences develop over time?

#### 3.1.1. Workload

**Figure 2:**
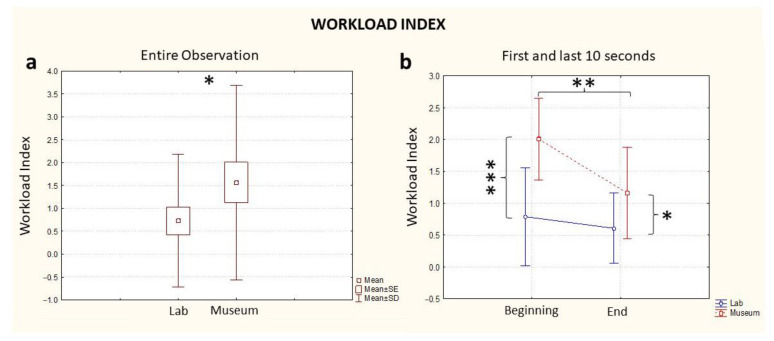
EEG Workload Index computed for the entire observation period (**a**) and during the first and last 10 s segments of the observation (**b**), in blue: the index computed in the lab and in red: the index computed in the museum. * stands for statistical significance at the level *p* ≤ 0.05; ** stands for statistical significance at the level *p* ≤ 0.01; *** stands for statistical significance at the level *p* ≤ 0.001.

The analysis of the entire observation revealed a higher level of Workload in the museum compared to the laboratory treatment (Location, *p* = 0.04, Figure 2a). When performing the ANOVA considering the variables Location (Museum/Laboratory) and Time (Beginning/End), this difference was visible from the beginning of the experience: if we consider the first 10 s of observation in both contexts, the museum environment induced a higher level of Workload in the participants (Time * Location, *p* < 0.001, Figure 2b, Beginning). At the end of the minute of observation, the museum still elicited a higher Workload level (Time * Location, *p* < 0.05, Figure 2b, End) but the difference with respect to the laboratory observation was lower than the beginning. There was also a statistically significant effect of the single variables Time (*p* = 0.01) and Location (*p* = 0.03) (Table 1). A further comparison between groups on the basis of the order of observation (lab first or museum first) was investigated, with no statistically significant differences found between groups for Workload values.

#### 3.1.2. Approach–Withdrawal

**Figure 3:**
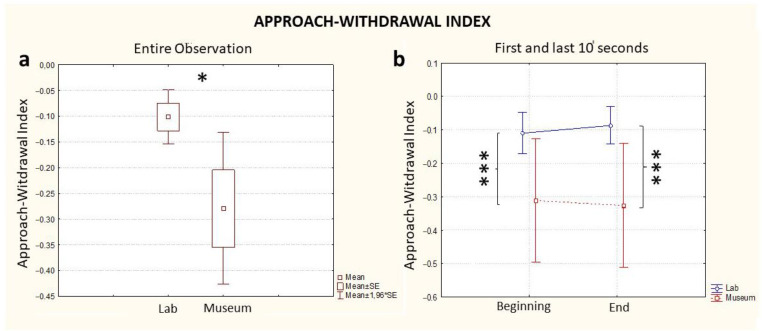
EEG Approach–Withdrawal Index computed for the entire observation period (**a**) and during the first and last 10 s segments of the observation (**b**), in blue: the index computed in the lab and in red: the index computed in the museum. * stands for statistical significance at the level *p* ≤ 0.05; *** stands for statistical significance at the level *p* ≤ 0.001.

The Approach–Withdrawal Index, considering the full time of exposure to the artifact, highlighted increased levels during the observation in the laboratory compared to the observation in the museum (Location, *p* = 0.03, Figure 3a). As with the case of the Workload assessment, a difference between the two conditions (museum vs. laboratory) was already visible in the first 10 s (Time * Location, *p* < 0.001, Figure 3b, Beginning) and was kept constant until the end of the experience (Time * Location, *p* < 0.001, Figure 2b, End). The effect of the single variables was statistically significant for Location (*p* = 0.02), but not for Time (*p* = 0.85) (Table 1). A further comparison between groups on the basis of the order of observation (lab first or museum first) was investigated, and no statistically significant differences between groups was found for Approach–Withdrawal values.

### 3.2. Emotional Index

**Figure 4:**
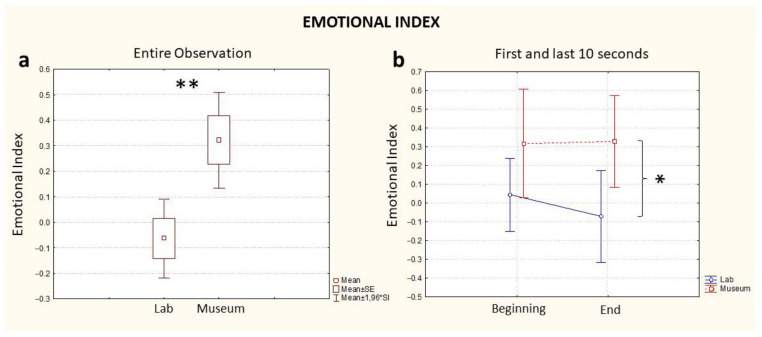
Emotional Index computed for the entire observation period (**a**) and during the first and last 10 s segments of the observation (**b**), blue: the index computed in the lab and red: the index computed in the museum. * stands for statistical significance at the level *p* ≤ 0.05; ** stands for statistical significance at the level *p* ≤ 0.01.

As with the previous indexes, considering the full time of exposure to the artifact, statistical analysis revealed a difference in the two conditions whereby the observation in the museum elicited a higher emotional response in the participants compared to the laboratory observation (Location, *p* = 0.03, Figure 4a).

Different from the Workload and Approach–Withdrawal indices, the comparison of the Emotional State Index computed for both conditions highlighted an absence of a difference at the beginning of the experience (Time, *p* > 0.05, Figure 4b, Beginning). However, such a difference was visible at the end of the observation, whereby the index computed during the museum observation registered a higher value than the index computed during observation in the laboratory (Time, *p* < 0.05, Figure 4b, End). The effect of the variable Location was statistically significant (0.01), but not for the variable Time (*p* = 0.06) (Table 1). A further comparison between groups based on the order of observation (lab first or museum first) was investigated, and no statistically significant differences were found between groups for Emotional Index values.

### 3.3. Behavioural Results

**Figure 5:**
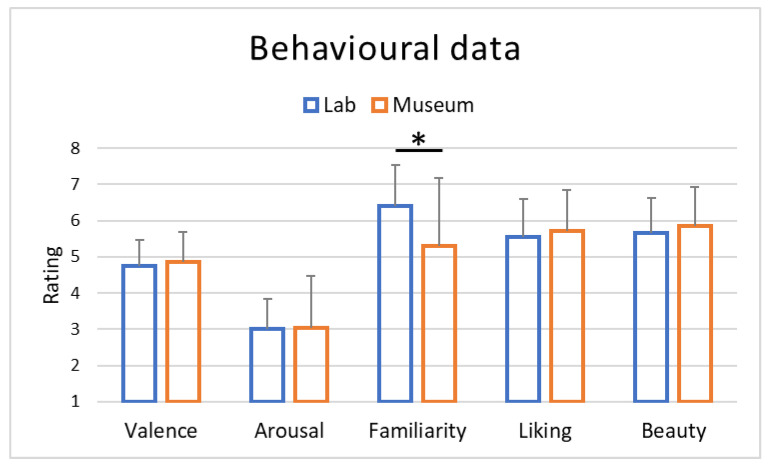
Averages of the behavioural results obtained through ratings (Likert scale 1–7) collected at the end of the entire art experience both in the lab and museum locations. * stands for statistical significance at the level *p* < 0.05.

At the end of each observation, participants were asked to rate their impressions about the dimensions of valence, arousal, familiarity, liking and beauty. Analysis of the explicit ratings highlighted substantial similarity between the two conditions (Table 2), with no statistically significant differences between the two location conditions except for familiarity (Figure 5). Indeed, only for the familiarity dimension were higher ratings observed for the lab condition compared to the museum condition (*p* = 0.03). Furthermore, among all correlations performed between neurophysiological indices and behavioral ratings, positive correlations were only observed for the familiarity and beauty dimensions with the average of the last 10 s of the artifact observation in the lab for the Approach–Withdrawal variable (respectively, *p* = 0.02, r = 0.51 and *p* = 0.04, r = 0.45).

## 4. Discussion

Results highlighted a general difference between experiencing the artwork in the museum or in the lab, suggesting a higher emotional involvement in the museum, also characterized by higher cognitive effort put into the art experience process in the natural environment. However, an increased level of withdrawal tendency was obtained in the museum in comparison to the lab, possibly reflecting a reaction to the experimental limits imposed on the experience in the real environment, and vice versa, a major acceptance of such limits when experiencing the artifact in the lab. Indeed, previous results highlighted the difference between real and experimental exposure to art, for instance in terms of free choice of viewing distance and time, which represents a core of the art experience [45]. Furthermore, in an in-lab experiment, an improved appreciation and enjoyment of artwork was recently found when employing EEG-based technologies during an art experience [46]. Moreover, an explanation for the higher withdrawal response to the session of the art experience in the museum could be due to the possible perception of a lack of technological applications seamless in the museum environment, which represents a major concern when designing technological solutions for museums [47]. An alternative explanation for the higher withdrawal in response to the session of art experience in the museum could be related to the spatial embodiment effect in VR [48], which is not possible in the museum. In short, the immersive impact of an archaeological artifact can simulate a high level of embodiment because of the scale and lack of barriers, such as those that exist in the museum showcase. At this point, the human interaction with the virtual model would intensify the embodied experience.

Furthermore, the relative higher approach tendency for the art experience in the lab compared to the one in the museum could be explained by the growing interest in VR technology employment in museum and artistic contexts in general [5,6,49] and recently, also specifically for archeological artifacts [50]. In particular, the effect of the environment on the art experience was supported by the evidence that the difference between the museum and the lab in terms of Approach–Withdrawal was maintained throughout the experience and was, in fact, confirmed when considering the beginning and the end of the art experience. It is also interesting to note that such kinds of VR solutions are also being developed for educational purposes, such as in the ArkaeVision Project, which combines VR and augmented reality in order to propose a more engaging and culturally qualified user-centered art experience [51]. However, when the purpose is educational, it is important to note that, at least in the context of a science lab, VR-mediated experiences could be more engaging but less fruitful in terms of educational performances, also requiring more cognitive effort by the user as indicated by an EEG-based workload assessment (calculated following a different formula from the one adopted in the present study) [52]. In the present study, however, we obtained an apparently opposite result, showing the museum condition to involve more cognitive effort compared to the lab one. This could be linked to the specific experimental conditions compared: in the aforementioned study by Makransky and colleagues, the compared experimental conditions were desktop display and head-mounted display (VR), whilst in the present study, the conditions were a real museum and an in-lab reproduction of the artifact by VR. Moreover, the theme of the experience was different between the Makransky and coworkers study and the present one: a science lab and an archaeological museum, respectively. This aspect does not allow a simple generalization of results between the scientific and humanistic framework of the studies, as indirectly suggested by a previous study where the humanistic or scientific background of the participants influenced their art experience (e.g. [53]). Such a difference between expert and not-expert participants was also shown to influence the dynamics of the art experience, in which the first and last seconds of observation elicited a different response in experts and not-experts [54,55]. The decrease in workload levels from the first 10 s to the last 10 s that occurred only in the museum condition—whereas for the lab condition the Workload levels were almost constant and always lower than the museum—could be explained by a physiological decrease in workload levels throughout the execution of the task in the case of tasks “more difficult to be processed”, such as the museum condition in comparison to the lab condition in the present study. This hypothesis has been suggested by a study from a different field of research, in which higher workload levels were estimated at the beginning of a task involving air traffic controllers and decreased toward the end, but only in the difficult condition, not in the easy one [56]. Additionally, research concerning the study of the behavior of visitors in real museums found an average observation of each artwork of 10 s [57], possibly corresponding to the beginning of the observation in our study in the museum condition and further supporting the relevance of such a time-lapse for a more engaging art experience. However, it is interesting to note that even though workload levels decreased from the beginning to the end of the task in the museum condition, they remained higher than the workload levels at the end timepoint, again supporting higher cognitive engagement represented by the ecological experience of the artifact as suggested above and also supported by Brieber and colleagues, who reported higher learning outcomes, in terms of memory recall, in the museum compared to the lab [58]. Moreover, it is worthy to note that the Workload Index employed here, which represents frontal Theta activity, has been reported as an index of processing and memory [21,59,60,61], thus in tight accordance with the aforementioned results of Brieber and colleagues. Concerning the key role exerted by the fruition environment, current models of aesthetic experience appear of high relevance, given their assumptions about the importance of the ecological factors [9,62].

Furthermore, the higher engaging potential of the museum condition in comparison to the laboratory one is also suggested from an emotional point of view, as shown by the present results concerning the Emotional Index. Indeed, the museum showed higher Emotional Index values than the laboratory condition, in particular at the end time-point at the conclusion of the entire art experience. This is in accordance with the evidence reported by Brieber and colleagues [58] of higher arousal perceived in real museums in comparison to the laboratory environment, despite these authors employing only declared data and not autonomic-based indices as in the present study. Ratings concerning the affective dimensions of arousal and valence were also investigated in our research, and no statistically significant differences were found between the museum and lab environments. Similar results were reported by Brieber and colleagues [58] and Szubielska and Imbir [63] for the valence dimension, but not arousal, which, as mentioned above, were higher in the museum context in their study but not in ours. This could be due to the kind of rating scale employed: in their studies, a six-point Likert scale (three negative and three positive) [58] and nine-point Likert SAM scale [63] were used, whilst in the present study, a seven-point Likert scale was used, allowing the central neutral rating and no reporting of figurative visual representations as in the SAM scales. This technical aspect could explain the different results obtained. Finally, when considering the beginning and end phases, the fact that the museum condition produced higher Emotional Index levels in comparison to the laboratory condition could be due to the slower nature of autonomic biosignals (order of seconds) compared to EEG (order of milliseconds), thus requiring a longer time to produce a clear physiological response to the environment.

Concerning behavioral ratings, the higher familiarity reported for the art experience in the lab could be explained by previous knowledge of the specific artifact, the Sarcophagus of the Spouses, mainly through technological means and not observation of the real statue. This is reasonable, due to the widespread popularity of the artifact among non-experts that are not used to visiting archaeological museums. Furthermore, the Sarcophagus of the Spouses was already the object of 3D reconstruction in an immersive environment [64], supporting the interest and relevance of the artifact although this is the first time in which the observation of it in the real museum was compared to a virtual reality observation in the lab.

Concerning the correlations between EEG-based indices and declared data, the correlation between Approach–Withdrawal in the last 10 s of observation and familiarity ratings, suggesting increasing approach tendency with increasing familiarity, could be explained by the processing fluency theory, which predicts higher liking linked to higher successful recognition of the stimulus [65]. This response has already been suggested in neuroaesthetics studies concerning poetry both for declarative [54] and EEG-based data [66]. Indeed, there was a link between ratings concerning liking and recognition of a poem’s excerpts [54] and also a correlation between event-related potential data (N400 and P600) and the ratings for aesthetic liking, supporting “perceptual-fluency-enhanced aesthetic liking” [66]. This is in accordance with the theory postulating that the higher the fluency the perceiver uses for processing an object, the more positive will be his or her aesthetic response, in particular with the Perceptual Fluency component of processing fluency, a component that is apparently increased by object familiarity [67]. Thus, cognitive fluency may influence the extent of linking of an artwork or an object [68], and the Approach–Withdrawal Index could be considered an index of implicit liking (or disliking) of a wide variety of stimuli; for instance, for figurative paintings [69] or music [70], and also in clinical settings [71] or for product design (for a review [72]).

The correlation between Approach–Withdrawal in the last 10 s of observation and beauty ratings, suggesting higher approach tendency in correlation with higher beauty ratings, could be explained by the recency effect; for instance, as shown for course evaluations by university students [73]. That is, observers would mainly rely on the last seconds of the art experience in order to express both an implicit (as indicated by the Approach–Withdrawal) and an explicit appreciation (indicated by the beauty rating). However, it is interesting to note that in a previous study investigating a Chinese sample of both artistic and commercial items, an approach tendency was found for artistic items irrespective of the judgement (or not) of the beauty of the item, while for commercial items there was an approach tendency for items judged as beautiful and a withdrawal tendency for those judged not to be beautiful [74]. This could be linked to the different culture and possibly also to the considered timing of exposure to the artwork, an aspect already suggested to be relevant for art fruition and response and probably involving the first 10-20 s of observation [55,75].

## 5. Conclusions

This study was part of an international collaboration between research institutions (Sapienza University and Duke University) and museums (the National Etruscan Museum of Villa Giulia, in Rome: the NeuroARTifact Project). The main goal of this project was to investigate and evaluate the cognitive impact of archaeological artifacts (empirical and digitally reconstructed) at different scales and through different technologies. Thus, portable EEG devices (computer-based), virtual and outdoor eye-tracking systems were used to acquire biometric data and virtual headsets to engender embodied simulation. The core research question concerned the investigation of neuroaesthetic experiences of material cultural and museum objects. The cognitive and neuroarchaeological study of an archaeological artifact should consider:

1. The study of the ancient mind (artifact as a cognitive product)
2. The study of ancient and modern «consumption» of art
3. The study of human embodiment as a learning experience.

The preliminary conclusions of this paper try to respond mainly to numbers two and three, while we expect to extend future research also to numbers one and two. The research protocol adopted was successful, from data acquisition data processing. 

More specifically, the results allowed us to respond to the research questions formulated above:

RQ1: What does the vision of the real funerary urn The Sarcophagus of the Spouses in a museum setting arouse compared to its reproduction in VR?

RQ2: How does the art experience differ over time in the museum or laboratory conditions?

RQ3: Is there a relationship between unconscious and self-reported perception?

In particular, concerning RQ1, the observation of the real artifact in the museum context appeared to be more exciting and more challenging, possibly providing a more complete art experience. The use of VR appears to be more engaging but not comparable to the live experience. The novelty factor of the visor could explain the interest shown by the participants during the virtual art experience and the higher level of embodiment.

The two conditions also differed in terms of their temporal dynamics (RQ 2), with the museum experience having a greater demand on cognitive resources in the first 10 s and a more intense emotional involvement in the final part of the observation, reflecting cerebral and autonomic dynamics proper of the employed biosignals and confirmed in the literature.

The unconscious perception appeared to be linked to declarative data only marginally, with a strong modulation by the recency effect. Such discrepancy between unconscious reactions and declarative data is well known in various fields such as consumer neuroscience [20,33,76,77,78,79,80,81,82,83,84,85], clinical [19,86,87,88,89] and operative environments [90,91,92], supporting the need for biosignal-based assessments.

This research on the neuroaesthetic experience in real and virtual environments shows, overall, the complexity of this phenomenology in just sixty seconds of an experiment, illustrated in Figure 2, Figure 3 and Figure 4. The observation of the sarcophagus generated different coefficients of cognitive and emotional reactions and we were able to track all the effects of these discontinuous “waves” in lab and museum environments. We certainly do not know the impact of “invasive” technological devices (VR headsets and EEGs) on the users/visitors, but this effect was standardized for the entire length of the experiment.

It was anticipated that the empirical impact of the artifact would be more pronounced in the museum. Yet the greater withdrawal in response to the art experience session in the museum compared to VR appears to demonstrate the relevance of the embodiment effect of VR, which is not achievable in the museum. The discovery or re-discovery of a virtual object at size and without physical hindrance (in the museum, people and exhibits) promotes a very holistic observation and reveals hidden aspects of the actual experience. In short, the user is solely concerned with the artifact’s spatial affordances. In fact, virtual embodiment can be defined by the sense of presence, the sense of agency and the sense of body ownership [48]. We can argue that in virtual reality, the environments’ details look more accurate but the whole experience is less engaging, as in this case, instead, in the museum. 

According to this viewpoint, there is no opposition between actual and virtual objects, and the virtual environment should be viewed as a hyper-real extension of the empirical experience and not a replica of reality. The interaction with the sarcophagus generates different stimuli in virtual and real models because they are observed at the same scale but in different contexts. 

It may seem premature to draw definitive conclusions from these preliminary data, which could benefit from an extension of the investigation to a longer duration of the art experience in order to assess reactions deriving from further elaboration of the processed information; for example, due to associative mechanisms [93]. However, there are reasons to hope that in the near future, neurophysiological studies can contribute to redesign museum collections in relation to the cognitive impact of their artifacts.

## Figures and Tables

**Table 1:** Tabulation reporting all formulas, calculations and obtained values concerning neurophysiological indices.

Index	Formula	Variable	Test	*p*-Value	Index Value
**Workload**	$\frac{\sum_{i=1}^{n}Frontal\;Theta}{n}$ Where n is the number of frontal electrodes	Location (Museum and Laboratory)	*T*-test	All: *p* = 0.04	Museum = 1.40 ± 4.03 Lab = 0.78 ± 3.07
		Time (Beginning. End)	*T*-test	Beginning vs. End (Lab): *p* > 0.05 Beginning vs. End (Museum): *p* < 0.01	Beginning = 1.41 ± 3.25 End = 0.92 ± 3.33
		Time * Location	ANOVA	Lab vs. Museum (Beginning): *p* < 0.001 Lab vs. Museum (End): *p* < 0.05	Museum Beginning = 1.88 ± 2.69 Museum End = 1.26 ± 3.81 Lab Beginning = 0.92 ± 3.56 Lab End = 0.55 ± 2.59
**Approach** **Withdrawal**	$\frac{\sum_{i=1}^{n}Frontal\;Alpha\;dx}{n}$ − $\frac{\sum_{i=1}^{m}Frontal\;Alpha\;sx}{m}$ Where n is the number of frontal right electrodes and m is the number of frontal left electrodes	Location (Museum and Laboratory)	*T*-test	All: *p* = 0.03	Museum = −0.25 ± 0.69 Lab = −0.09 ± 0.27
		Time (Beginning. End)	*T*-test	Beginning vs. End (Lab): *p* > 0.05 Beginning vs. End (Museum): *p* > 0.05	Beginning = −0.20 ± 0.65 End = −0.18 ± 0.63
		Time * Location	ANOVA	Lab vs. Museum (Beginning): *p* < 0.001 Lab vs. Museum (End): *p* < 0.001	Museum Beginning = −0.29 ± 0.83 Museum End = −0.27 ± 0.80 Lab Beginning = −0.11 ± 0.29 Lab End = −0.08 ± 0.26
**Emotional** **Index**	|SCL| ∗ HR	Location (Museum and Laboratory)	*T*-test	All: *p* = 0.03	Museum = 0.32 ± 0.88 Lab = −0.06 ± 0.72
		Time (Beginning. End)	*T*-test	Beginning vs. End (Lab): *p* > 0.05 Beginning vs. End (Museum): *p* > 0.05	Beginning = 0.18 ± 1.10 End = 0.13 ± 1.13
		Time * Location	ANOVA	Lab vs. Museum (Beginning): *p* > 0.05 Lab vs. Museum (End): *p* < 0.05	Museum Beginning = 0.32 ± 1.27 Museum End = 0.33 ± 1.07 Lab Beginning = 0.04 ± 0.85 Lab End = −0.07 ± 1.07

* stands for the interaction between variables.

**Table 2:** Tabulation reporting all values and results concerning behavioural data.

Dimension	Lab	Museum	*p*-Value (Wilcoxon)
Valence	4.75 ± 0.71	4.86 ± 0.83	0.36
Arousal	3.00 ± 0.85	3.04 ± 1.43	0.81
Familiarity	6.40 ± 1.14	5.31 ± 1.86	0.03
Liking	5.55 ± 1.05	5.72 ± 1.12	0.31
Beauty	5.65 ± 0.98	5.86 ± 1.08	0.37

## Data Availability

Data will be made available on reasonable request from Fabio Babiloni (fabio.babiloni@uniroma1.it).
